# Non-surgical endodontics: contemporary biomechanical preparation of the root canal system

**DOI:** 10.1038/s41415-025-8599-1

**Published:** 2025-04-11

**Authors:** Phillip L. Tomson, Nick Adams, Damian Kavanagh, Satnam Singh Virdee

**Affiliations:** 756111126641109609304 Senior Clinical Lecturer and Honorary Consultant in Restorative Dentistry, University of Birmingham School of Dentistry, College of Medicine and Health, 5 Mill Pool Way, Edgbaston, Birmingham, B5 7EG, United Kingdom; 026513434131162669533 Specialist Endodontist, Part-Time Clinical Lecturer in Restorative Dentistry, University of Birmingham School of Dentistry, College of Medicine and Health, 5 Mill Pool Way, Edgbaston, Birmingham, B5 7EG, UK; Madeley Dental Practice, 69 High Street, Madeley, Telford, Shropshire, TF7 5AU, United Kingdom; 936158463105481081861 General Dental Practitioner, Level 2 Accredited Endodontics Practitioner and Part-Time Clinical Lecturer in Restorative Dentistry, University of Birmingham School of Dentistry, College of Medicine and Health, 5 Mill Pool Way, Edgbaston, Birmingham, B5 7EG, UK; Barnt Green Dental, 111 Hewell Rd, Barnt Green, Birmingham, B45 8HT, United Kingdom; 395332027280436257512 Clinical Lecturer and Honorary Speciality Registrar in Restorative Dentistry, University of Birmingham School of Dentistry, College of Medicine and Health, 5 Mill Pool Way, Edgbaston, Birmingham, B5 7EG, United Kingdom

## Abstract

Treatment of apical periodontitis requires disinfection of the root canal system through means of biomechanical preparation. This is achieved by a process of shaping the root canal space while simultaneously using antibacterial agents to reduce microbiological load in the root canal system in order for inflammation of the periradicular tissues to resolve and the body to heal itself. The fundamental principles underlying this process have not changed in decades; however, in contrast, the armamentarium available to the clinician continues to evolve rapidly. Nickel-titanium file design has made a significant step forward in the last ten years. Areas of development have focused on the metallurgic properties, motion of the instrument and cross-sectional design. The resulting contemporary designs have allowed the clinician to manage more complex root canal systems more predictably in situations which would otherwise have proved difficult using conventional techniques; effectively, it has made it easier to prepare a root canal. These newer systems also require fewer instruments to prepare a canal and some, which have adopted a reciprocating (rotational) motion, may only require one engine-driven file. Significant energy has been devoted to attempting to enhance efficacy of irrigant activity using different techniques. Although this has shown promise in *in vitro* studies, this has yet to be shown to translate to an improved clinical success rate based on comparative clinical studies performed. Contemporary biomechanical techniques used to clean and shape the root canal system should result in improved confidence and predictability when managing endodontic disease.

## Key points

- Providing a root filling with no voids that extends within 2 mm of the radiographic apex and has a satisfactory coronal restoration are the most important factors that an operator can control to significantly improve the outcome of root canal treatment.
- Determining the size of the canal early in treatment suggests a safe strategy of negotiation to make the small/difficult canal into a larger, easier one to manage.
- Improvements in nickel-titanium file design have made it easier to prepare root canals.

## Introduction

Apical periodontitis is most frequently caused by infection or injury to the pulp tissues leading to pulp necrosis and subsequent bacterial colonisation of the root canal system.^1^^,^^2^ Success of root canal treatment to prevent or cure apical periodontitis is reliant upon accurate diagnosis and performing each stage of treatment to a high standard. The basic strategy to treatment has not fundamentally changed for decades and it is aimed at reducing bacterial load in the root canal system and preventing reinfection by providing a seal throughout the canal to the coronal restoration. Four factors significantly improve outcome of primary root canal treatment:^3^

- Pre-operative absence of periapical radiolucency
- Root filling with no voids
- Root filling extending within 2 mm of the radiographic apex
- Satisfactory coronal restoration.

**Fig. 1:**
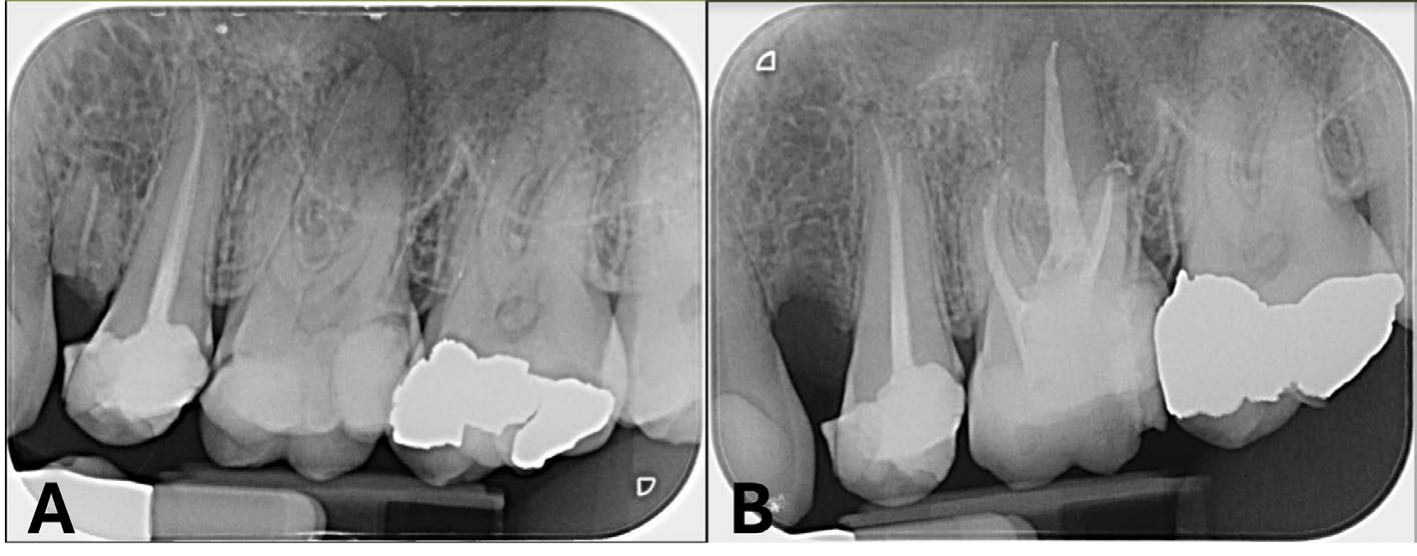
a) Preoperative periapical radiograph of left maxillary first molar with symptomatic apical periodontitis. b) Postoperative radiograph showing root canal treatment achieving technical operative factors determined by Ng *et al.* 2008 which significantly improve the outcome of primary root canal treatment. Case displayed was carried out by Shruti Panchani (undergraduate student at Birmingham School of Dentistry)

The operator cannot influence the first factor but must highlight this to the patient when discussing the overall prognosis; however, it is possible to have control over the remaining three factors (Fig. 1). Over the last 30 years, there have been huge developments in the armamentarium available for executing root canal treatment, with the use of nickel-titanium (NiTi) files, a wider variety of instruments to help find and negotiate root canals, and the use of methods to improve the efficacy of irrigants. Although the procedures have improved and arguably make cleaning and shaping of the root canal system simpler, the basic strategy remains the same.

Herbert Schilder's seminal paper entitled ‘Cleaning and shaping the root canal'^4^ has been adopted as the ideological approach to managing the infected root canal system. In this, it refers to cleaning and shaping as ‘the removal of all organic substrate from the root canal system and the development of a purposeful form within each canal for the reception of a dense and permanent root canal filling'. For ease of recall in the discipline, this is commonly abbreviated into ‘clean', ‘shape' and ‘fill'. Schilder established these objectives which can be considered independently as the biological and mechanical objectives of root canal preparation.

## Biological objectives of cleaning and shaping the root canal system

**Fig. 2:**
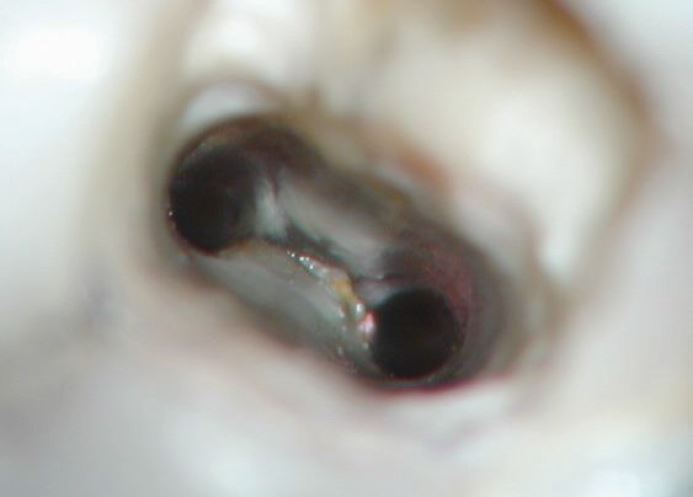
Biofilm located in the isthmus between distal buccal and lingual canals of a mandibular first molar seen under operating microscope during retreatment procedure

Pulp necrosis renders the root canal space unprotected, providing an ideal environment with an abundant nutrient source to support microbial proliferation. If undisturbed, such a space allows microorganisms to multiply and mature, subsequently developing a particular pathogenicity that can induce inflammatory disease of the periradicular tissues. Primary endodontic infections are usually caused by opportunistic oral bacteria that proliferate in the root canal space. Endodontic microbiota exist as dense bacterial aggregates/coaggregates, or in a fluid phase adhering to canal walls forming multi-layered communities that resemble biofilm (Fig. 2). The root canal space is a complex environment with anatomy unique to every tooth, with such features as accessory canals, isthmi, branches, fins and other aberrant forms which can be found in any part of the canal. It is not possible to mechanically prepare/clean the whole of the root canal system^5^ and therefore other methods of cleaning this abstract space is required. The biological objectives suggested by Schilder^4^can be summarised as:

- Disinfect as much of the root canal system as feasible
- Remove potential nutrient sources that could support recolonisation of microorganisms into the root canal system
- Block recontamination of the root canal system.

## Mechanical objectives of cleaning and shaping the root canal system

Cleaning and shaping are procedures which are not independent of one another. Altering the shape of the root canal system with different endodontic instruments facilitates cleaning in two ways: 1) direct removal of bacteria and nutrient sources from the root canal system; and 2) facilitates antibacterial agents to penetrate deeper into the root canal system to enhance disinfection. Schilder^4^ outlined design objectives to shape the canal facilitating cleaning but to also produce a shape that would aid obturation. The mechanical objectives can be summarised as:

- A continuously tapered preparation should be produced
- The canal axis should be maintained in the centre of the root
- The original position of the foramen should be maintained and it should not be enlarged.

A continuously tapering preparation is required to deliver a chemically active irrigant to the canal terminus^6^^,^^7^ in order to remove and destroy microorganisms that are driving the inflammatory process in periradicular tissues. Adequate space is required in order for solutions to pool coronally so they can be delivered to the apical portion of the canal. It has been proposed that the apical preparation should be prepared to larger diameters.^8^^,^^9^^,^^10^ Such an approach may lead to destruction of the fragile structure of the apical foramen and thus complicate the chance for an optimal apical seal. There is also greater chance of apical perforation^11^ and worsening the prognosis of the tooth.

## Technical phases in cleaning and shaping the root canal system

In most cases, initial treatment of periradicular disease is via an orthograde approach to access the root canal system through the crown, aiming to disinfect dentine in close proximity to the inflamed periradicular tissues. Access is a dynamic process and can be returned to at any stage of root canal preparation. The aim of coronal access is to remove the pulp roof and provide an unimpeded pathway for instrumentation of all parts of the root canal system. Failure to carry out satisfactory coronal access can lead to difficulty in locating and negotiating root canals. This can result in complicating the ability to shape and clean the root canal system. The technical stages of access are not considered in this article and have been reviewed in detail elsewhere.^12^ Preparation of the root canal system can be considered in the following phases:

- Initial canal negotiation
- Coronal enlargement
- Apical exploration and completion of glidepath
- Length determination and apical patency
- Canal preparation.

### Initial canal negotiation

The initial coronal exploration will immediately give the operator an idea of how easy or challenging a case may be and determine what strategy for managing the case should be. It should be carried out with small stainless-steel hand files eg an ISO size 8 or 10. The ease and depth of advancement gives important information regarding size of the root canal and depending on how the file advances, the canal can be classified into small, medium, or large (Table 1). Classifying canals as such provides a practical framework to highlight challenges, guide technique and ensure safety and efficiency.

**Table 1:** Basic canal classification in order to inform strategy of negotiation/preparation of the canal

	**Small**	**Medium**	**Large**
Definition	Size 8/10 file does not advance into the canal very easily and it is difficult to get into the apical region	Size 8/10/15/20 advance easily into the canal and reach the apical region passively without going beyond the end of the canal	Size 40+ pass easily into the apical region passively
Resulting management strategy	These canals often require careful negotiation and initial management	These canals are more straightforward to manage and allow for standard rotary instrumentation after glide path preparation	These canals often have a wider diameter and may require less aggressive preparation to avoid over-instrumentation

### Coronal enlargement

Early coronal enlargement is key to facilitate a pathway to the more anatomically complex apical third of the canal. This has several advantages (Table 2).

**Table 2:** An outline of mechanical and biological advantages of coronal enlargement

**Mechanical**	**Biological**
Reduces radius of the curve	Increased coronal irrigation
Decreased stress on instruments	Decreased risk of inoculating bacteria apically
Stable reference point	Decreased risk of packing debris apically
Decreased risk of procedural errors	Deeper insertion of irrigating needle
Improved tactile feedback	
Improved vision	

Initial canal enlargement for a small canal is done by hand filing using a systematic approach (ISO size 6, 8, 10) in a watch-winding fashion. Larger stainless-steel hand files can be used subsequently (15, 20, etc) to follow a traditional pre-flare technique. The key at this stage is not to be ‘greedy'; take what the canal will give you but no more, then increase the size of the file. If that feels too tight, go back to a smaller file and work it further to make room for the next file. One may go back and forth a little through these files but make sure the sequence is followed and never miss an instrument. Throughout this process, irrigate and recapitulate thoroughly between each instrument. This has colloquially been called the ‘endo dance' and can be time-consuming in tight canals.

**Fig. 3:**
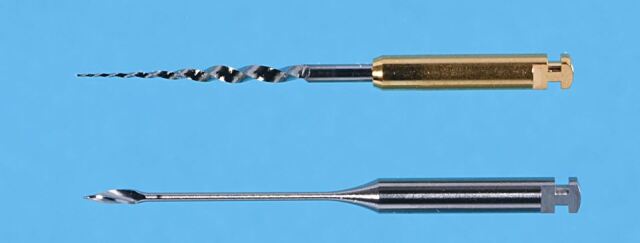
Burs designed for radicular access (top to bottom): Protaper Next XA and X-gates

**Fig. 4:**
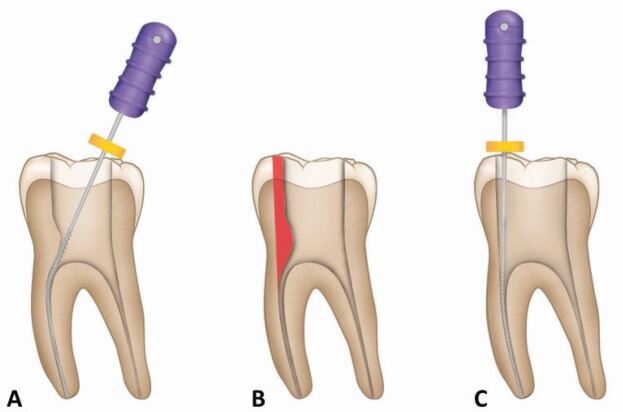
a, b, c) Developing straight line access - the process of removing coronal and radicular dentine (coronal third) in order to prevent deflection of the file from a straight path until it penetrates deeper into the canal. File is deflected by coronal and radicular third dentine highlighted in panels b and c. This dentine is removed and the file passes deep into the canal along a straight path without deflection (image reproduced with permission from the British Endodontic Society)

In a small canal, the initial aim is to enlarge the orifice enough to make way for engine driven instruments, such as a ProTaper Sx or Gates Glidden burs to then rapidly advance this stage (Fig. 3) and straighten up any coronal curvatures (Fig. 4).

### Apical exploration and completion of glidepath

Apical negotiation and glide path creation are essential for establishing a safe and efficient pathway for subsequent instrumentation and can frequently be the most difficult stage in root canal therapy. The narrow anatomy of some root canals, particularly in calcified or curved cases, makes apical exploration complex. Without precise technique, the operator may inadvertently create complications, such as:

- Blockages - accumulation of dentin chips that prevent file progression
- Ledges - deviation from the canal's original path due to improper file handling
- Perforations - instrument breaching the canal walls, which can compromise the tooth's prognosis.

In order to prevent iatrogenic errors, the operator should maintain a methodical approach and follow the same principles outlined above.

## Steps for effective glide path creation

1. Initial negotiation with hand files:

Begin with small stainless-steel hand files (sizes 6, 8, and 10). Work these files until they become loose in the canal, establishing a preliminary pathway to the apical terminus

2. Progressive enlargement using engine-driven instruments:

Once the pathway is initiated to an ISO size 10/15 hand file, an engine-driven NiTi file can be used to aid glide path creation in a safe and efficient manner with minimal risk of transportation. There are a huge number available and some have been highlighted in Table 3.

**Table 3:** Summary of examples of engine driven NiTi files to aid glidepath development based on prominent design feature (taper design and motion)

**Continuous taper**	**Variable taper**	**Reciprocation**
PathFile (Dentsply Sirona, Maillefer, Switzerland)	ProGlider (Dentsply Sirona, Maillefer, Switzerland)	WaveOne Gold Glider (Dentsply Sirona, Maillefer, Switzerland)
HyFlex EDM (Coltene Altstätten, Switzerland	TruNatomy Glider (Dentsply Sirona, Maillefer, Switzerland)	R-Pilot (VDW, Munich, Germany)
	G-Files (Micro-Mega, France)	
	ProTaper Ultimate Slider Glider (Dentsply Sirona, Maillefer, Switzerland)	

The ProGlider instrument as an example (Fig. 3) can be used alone for glide pathway management; however, other systems suggest the use of more than one instrument, such as PathFiles. After initial opening of a tight canal with small stainless-steel hand files (6, 8, 10), by working each one loose before using the next (working each file loose), the length navigated to by the size 10 file can be transferred to the ProGlider instruments. The ProGlider can be worked to this length and will progressively open the canal due to its increasing percentage tapers from 2% to 8%.

When full working length cannot be initially achieved, the space created by the ProGlider will allow the small stainless-steel hand files (6, 8, 10) to penetrate deeper into the canal and negotiate to length. The ProGlider can then secure the glide path.

### Length determination and apical patency

Determining the canal length is fundamental to the process of root canal preparation and this can be achieved through many processes, which include radiographs, tactile discernment, a paper-point approach, and with the use of an electronic apex locator (EAL). It is widely considered that the EAL is a mandatory instrument in the dentist's armamentarium. Among manufacturers of these electronic devices, there is not always consistency about which anatomical point the EAL will identify. It is recognised by users that the most reliable reference point, regardless of the device, is the zero reading. It is frequently misunderstood, but by definition, to use an EAL correctly, the canal has to be patent.

**Fig. 5:**
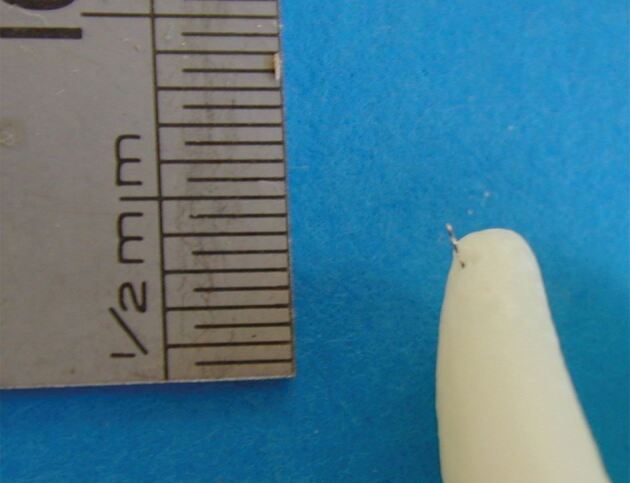
Photograph of root-end of extracted tooth with small file through the foramen, demonstrating the foramen can be distant from the tip of the root (radiographic apex)

Modern apex locators are so reliable that they represent a significant improvement over traditional radiographs in determining the length of the root canal. Radiographs cannot reliably account for the variability in the position of the apical foramen relative to the radiographic apex, which may differ by as much as 0-3 mm (Fig. 5). With an EAL, the operator can accurately determine the endpoint of a canal and confidently complete precise preparation and obturation. In a prospective observation study of primary root canal treatment, it was demonstrated that obturation 0-2 mm from the zero reading achieved the highest success rate (85.8%). Short-root fillings (>2 mm) (74.3%) were next and long-root fillings had the worst success rate (67.1%).^13^

Apical patency is a technique whereby the apical extent of the canal is maintained free of debris by a small file which passes through the apical foramen. In principle, once a glidepath has been established, a small hand file (ISO 6, 8, 10) is passed slightly beyond the foramen by 1 mm by the recapitulation file after each instrument is used to shape the canal. A patency technique can reduce the risk of debris accumulation in the apical portion of the canal and decreases the risk of procedural error.^14^ It is also suggested that the mechanical action of a patency file may help remove bacteria that are present in the apical constriction and foramen.^15^ It must be stressed that when using a patency technique, only small instruments should be used and should not extend greater than 1 mm beyond the foramen. Abuse of this principle can lead to over-instrumentation, enlargement/transportation of the foramen, extrusion of infected debris and mechanical damage to the apical tissues.

It has been considered that using a patency technique can result in negative effects, such as extrusion of debris into the apical tissues and increased risk of postoperative pain.^16^ A recent prospective randomised trial demonstrated that maintenance of patency during biomechanical preparation had no significant influence on postoperative pain.^17^ The patency technique is now well-established and has shown that if achieved, the odds of success can be doubled, and is a significant intra-operative factor in achieving periapical health following root canal treatment.^13^

### Root canal preparation - a contemporary approach

Once a glide path patency and the working length of the canal have been established, the remaining part of the root canal preparation should be straightforward. There are countless methods of preparing the remainder of the root canal with the use of stainless-steel hand files, ultrasonic instruments and most commonly, engine-driven NiTi instruments. Evidence-based recommendations from the European Society of Endodontology's (ESE's) S3 guidelines recommend that root canal preparation using contemporary engine-driven techniques with NiTi instruments should be used for managing apical periodontitis and any tested NiTi instrument is appropriate.^18^ Current advancements have concentrated on engine-driven NiTi instruments and therefore will be the priority of the next part of this paper.

Nitinol was proposed as a material for root canal instruments in 1988^19^ due to its increased flexibility compared to stainless steel. Since then, there has been considerable progress in instrument design using NiTi alloys over the last four decades. Early NiTi file designs adopted an approach with fixed tapers and passive cutting file flutes with radial lands. To enhance ease of use, safety and efficiency, the following design features have been given most attention: variable tapers, cutting flutes, the material, cross section and the motion. Variable tapers limit the surface area of the cutting portion of the file to a specific point within the canal and cutting flutes improve efficiency. More recently, developments have been seen in the motion of novel cross-sectional designs and metallergy.

**Fig. 6:**
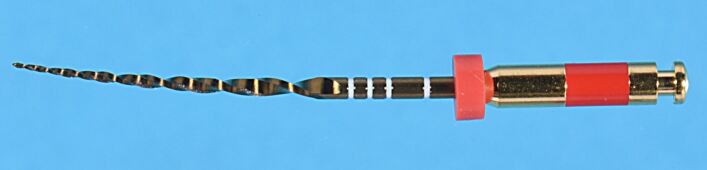
WaveOne Gold primary (25, 0.07) instrument is pre-curved off central axis without permanent deformation due to reduced shape memory compared to conventional NiTi instruments

Several novel thermomechanical processing and manufacturing technologies have been introduced to optimise the microstructure of NiTi alloys to improve flexibility and fatigue resistance of endodontic instruments. NiTi files such as Reciproc Blue (VDW, Munich, Germany), WaveOne Gold (Dentsply Sirona, Ballaigues, Switzerland), ProTaper Gold (Dentsply Sirona, Maillefer, Switzerland) are produced using these procedures. The precise details of the thermomechanical processing remains unknown due to the commercial sensitivity; however, these modifications have significantly improved flexural fatigue resistance compared to files made from conventional NiTi alloy. An interesting feature of this alloy is reduced shape memory at room temperature, which demonstrates less bounce back in comparison to traditional NiTi files. This makes these files feel rubbery when flexed compared to the immediate bounce back to their original shape of other NiTi instruments. This means it is possible to lightly pre-curve the instrument off the central axis of the instrument without permanent deformation (Fig. 6), which can be a real advantage during placement into difficult-to-reach canals. The differing colours result from differing thicknesses of titanium oxide being created during the heat treatment.

The use of NiTi instruments in a continuous rotating motion has been the mainstay of engine-driven file design. Reciprocating motions have been more recently developed as a new technique to prepare the root canal. Although reciprocating motions have been used before - the Cursor Filing Contra-Angle in 1928 (W&H, Austria), the Racer in 1958 (W&H, Austria) and the Giromatic in 1964 (Micro-Mega, France) - these reciprocating files had equal clockwise (CW) and anticlockwise (ACW) motions and therefore never made a complete 360 degree rotation during the movement sequence. In 2008, Ghassan Yared^20^ suggested a single-file technique with a reciprocation motion. This had an unequal CW and ACW movements usinga F2 ProTaper instrument (Dentsply Maillefer, Switzerland) with an ATR electronic motor (ATR, Italy). Dentsply International introduced two reciprocating (rotational) systems based on this concept: Reciproc (VDW, Germany) and WaveOne (Dentsply Sirona, Maillefer, Switzerland). The principle behind the reciprocation technique is that there is a rapid repetitive process of engagement and disengagement of the file with the canal wall through the precise CW and ACW movement. As these motions are not equal, the file will effectively rotate through 360 degrees after a particular number of phases of motion. This decreases load on the file and permits it to follow the canal more easily. The technique proposes that, in most cases, only one reciprocating (rotating) file is required to prepare each canal.

Other developments in file design include:

1. An off-centred cross-section which is used in the following instrument systems: 2Shape (Micro-Mega, France), WaveOne Gold, ProTaper Next (Dentsply Sirona, Maillefer, Switzerland) and ProTaper Ultimate (Dentsply Sirona, Maillefer, Switzerland). The principle of this design is to reduce surface area of contact between the file and the canal wall (Fig. 7), reducing the load on the file. This cross-section also allows room for debris to accumulate between the flutes of the file and driven in a coronal direction. It is expected that such a feature will enhance cleaning of the root canal system due to efficient debris removal. The off-centred cross-sectional design also allows greater flexibility in the file and creates a larger envelope of motion and will cut a larger preparation compared to a file with a conventional cross-section design

**Fig. 7:**
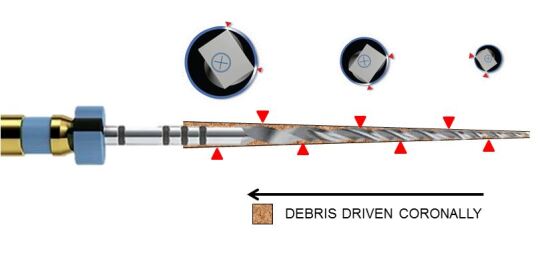
ProTaper Next file design with rectangle off-set cross-section along its length which results in only two points of engagement (red arrows). In principle, this creates space for debris to be driven coronally when in motion
2. Files with triangular or trapezoidal cross-sections enhance cutting efficiency and reduce engagement with canal walls, as seen in EdgeFile X7 (EdgeEndo, USA) The broader base of the trapezoid provides strength and resistance to cyclic fatigue, while the narrower apex maintains cutting precision
3. Files with offset centre of rotation is seen in the XP-endo Series (FKG Dentaire), minimising canal binding and improved debris removal efficiency.^21^ These files are highly flexible and expand at body temperature to adapt to irregular canal anatomies, ensuring optimal cleaning of complex root canal systems. This design allows for preservation of tooth structure, addresses anatomical complexities that traditional NiTi files struggle with, and as the files adapt to canal curvature, minimise procedural errors, such as canal transportation, ledging, or perforation.^22^ Overall, these innovations aim to improve the precision, flexibility and safety of root canal preparation.

**Fig. 8:**
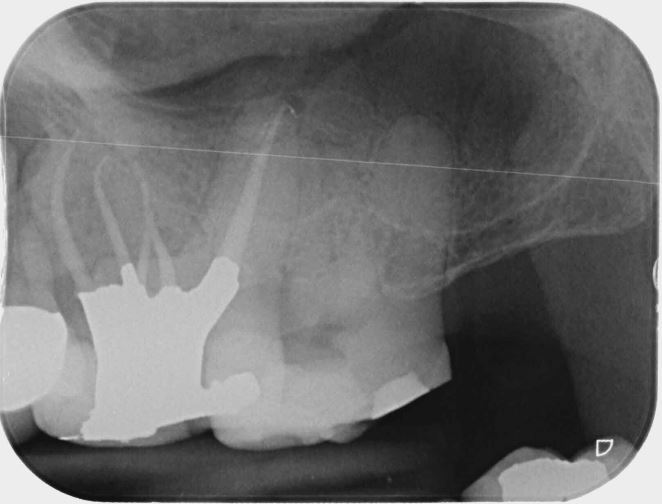
Postoperative periapical radiograph of root treated upper left first molar showing a ‘skinnier' style of preparation than previously produced

In an age of minimally invasive dentistry, there is a drive to preserve tooth tissue, and root canal preparation has not been overlooked. Several manufacturers have decreased the diameter of their wire used to fabricate endodontic NiTi files from 1.2 mm to 1.0 mm. This has led to the preservation of more coronal dentine and an evolution of canal shapes appearing ‘skinnier' than previously (Fig. 8). The advantage of this is that more pericervical dentine is preserved, which is crucial for the long-term mechanical integrity of the tooth.

## Irrigation

### Functions

Endodontic irrigation serves several critical and dynamic functions throughout the bio-mechanical debridement of root canals^23^ (Table 4). Upon immediately accessing the pulp chamber, irrigant solutions flush out gross dentinal debris created from cutting instruments to improve visualisation. During subsequent canal exploration and preparation, their primary function changes to lubricate canals, dissolve residual organic matter and provide antibacterial activity.

**Table 4:** Summary of desired properties from an irrigant solution used in endodontics

Biological	High bactericidal efficacy against microorganisms in biofilms and their planktonic state Inactivate endotoxin Non-toxic and hypoallergenic to vital tissues
Mechanical	Flush out debris Lubricate the canal
Chemical	Dissolve organic tissue Dissolve inorganic tissue and remove smear layer

### Solutions

At present, no single irrigant solution possesses all of the desirable properties necessary to fulfil the aforementioned functions (Table 4). National surveys have demonstrated a range of solutions being taught to undergraduates and used in general practice settings, with some aspects having stronger consensus than others.^24^ Of the various solutions listed, three warrant further discussion.

#### Sodium hypochlorite

Sodium hypochlorite (NaOCl) universally remains the most popular irrigant solution to date and is considered the gold standard.^25^ This is because of its broad-spectrum antimicrobial properties and ability to digest organic matter.^26^ While the strength reported in the literature ranges from 0.5-6.0%, concentrations of ≤3% are generally advocated to offset its cytotoxic potential, which can cause a severe physiological response if extruded through the apical foramen into anatomical compartments.^24^ While these strengths possess less chlorine ions and thus may be less efficient at dissolving organic matter, this can be offset with frequent flushing. Sodium hypochlorite is incapable of eliminating the inorganic component of the smear layer, which in itself harbours microorganisms. Despite these limitations, NaOCl still remains the primary irrigant solution used throughout the duration of the procedure.

#### Ethylenediaminetetraacetic acid

Ethylenediaminetetraacetic acid (EDTA) remains the second most popular irrigant of choice and is often used as a secondary irrigant in conjunction with NaOCl.^24^ This is primarily due to its chelating mechanism of action which is used to eliminate the inorganic component of the smear layer. This 1-2 μm thick granular layer formed following mechanical instrumentation penetrates dentinal tubules and remains adherent to canal walls.^27^ Its presence compromises disinfection by harbouring microorganisms and necrotic debris, restricting irrigant penetration^28^ and providing avenues for microleakage following canal filling.^29^ EDTA is thus used as a penultimate rinse to NaOCl, with this sequence demonstrating a significant prognostic factor in the healing of periapical lesions in retreatment cases.^13^ Periods of EDTA exposure less than five minutes (ideally one minute) are currently recommended for this purpose so as to avoid increases in dentinal erosion and reductions in dentine microhardness, which could theoretically lead to increased risks of tooth fracture.^30^^,^^31^

#### Chlorhexidine digluconate

Aqueous chlorhexidine digluconate (CHX) delivered at 2% strength (not the 0.2% available as a mouthwash) is a broad-spectrum antimicrobial solution which is effective as a root canal irrigant against a broad range of gram-negative and positive bacteria.^32^^,^^33^ This is in addition to notable anti-fungal activity against common oral commensals, namely *Candida albicans*, that invade into the root canal.^34^ It has been suggested as a final rinse as it is adsorbed into dentine and can show residual antimicrobial activity up to 12 weeks.^35^ Users of CHX irrigant should be mindful that if combined with NaOCl, an orange-brown precipitate can form, which can contain a cytotoxic substance called para-chloroaniline (PCA). PCA has been shown to be carcinogenic in animals and although it has not been reported as a consequence with its use in dentistry, the use of both irrigants in the same case should be completely avoided.

### Conventional irrigation

**Fig. 9:**
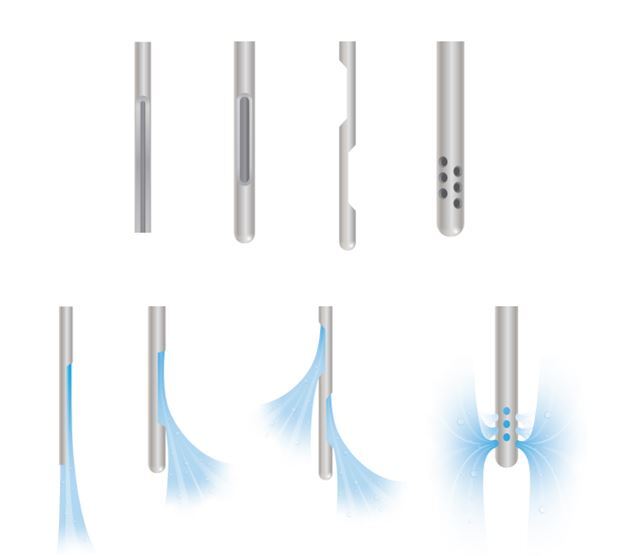
Various safe-ended irrigation needles that direct fluid flow laterally from the needle end and dissipate fluid pressure to help avoid rapid expulsion of the fluid in what would be an apical direction when in the canal (image reproduced with permission from the British Endodontic Society)

Paradoxically, the more effective the canal has been irrigated, the higher the risk of extrusion. Conventional endodontic irrigation should thus be a balance between achieving deep irrigant penetration and avoiding accidental extrusion. Key to this equilibrium is the design of the irrigating needle with open-ended designs (Fig. 9), and larger gauges that are more likely to bind to canal walls more coronally being discouraged. Alternatively, smaller gauges of 27 and 30 with side-vented, luer-lock designs offer operators a safer way to disinfect the apical canal more effectively.^36^ This should be achieved with light finger pressure with constant motion of the needle tip, which should not be advanced more than 2 mm from the working length. Sophisticated irrigant dynamics studies have demonstrated this is the distance in which irrigant solutions are expressed past the tip of the needle.^37^ Operators can choose to bend the needle or place rubber stoppers at this length to help maintain length discipline while irrigating the canal. A flexible polyethylene needle, IrriFlex (Produits Dentaires, Vevey, Switzerland), has been produced and has interesting features. It is suggested to have good penetration into the canal, can conform to the curvature of the canal and delivers irrigant at a high flow rate through two side vents.

### Enhancing irrigant activity

**Fig. 10:**
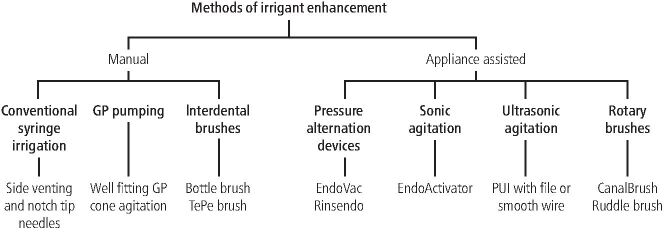
Summary of manual and appliance assisted techniques irrigant enhancement methods

In certain cases where mechanical instrumentation is limited, such as those with complex root canal anatomy, operators must rely more heavily on the chemical component of the disinfection strategy. To date, there have been several hand and mechanical agitation techniques adopted to enhance irrigant activity (Fig. 10). *In vitro* studies have demonstrated these techniques to be more efficacious than conventional needle irrigation at eliminating residual dentinal debris, the smear layer and achieving deeper tubular penetration.^38^ Interestingly, manual dynamic activation or gutta-percha point pumping has shown to be as effective as more sophisticated techniques at achieving smear layer and dentinal debris removal from within root canals.^39^ This insight is based on *in vitro* studies, and clinical comparative studies show little difference in clinical efficacy and effectiveness between conventional irrigation and adjunctive techniques used to enhance irrigation effectiveness. The recently published ESE S3 guidelines ‘suggest not to use adjunct therapy in addition to traditionally syringe-needle-based irrigation'.^18^

A novel irrigant agitation technique using multisonic sound waves called GentleWave (Sonendo Inc, USA) system has shown recent interest. The philosophy of the device is to enhance cleaning efficiency so that access cavities can be reduced in size, allowing dentine to be more readily preserved. It also makes root canal treatment easier and compromises the structural integrity of the tooth less. *In vitro* studies have been promising, showing that tissue dissolution is significantly faster with the GentleWave system compared to conventional irrigation devices.^40^ Other interesting areas of development to enhance irrigation include laser-activated irrigation. Lasers can be applied to water or irrigants to transfer energy through cavitation, leading to intense liquid dynamics for enhanced mechanical cleaning. Examples include a Er:YAG laser which develops photon-induced photoacoustic streaming and another approach uses Er,Cr:YSGG lasers (waterlase) which is delivered much deeper into the canal.

The ESE S3 guidelines^18^ suggest to use a combination technique of NaOCl and EDTA and based on this, a pragmatic evidence-based irrigant protocol would be as follows:

- 1−5.25% NaOCl throughout duration of canal exploration and preparation with frequent rinsing
- 17% EDTA penultimate rinse <5 minutes after canal preparation
- 1−5.25% NaOCl final rinse.

When biomechanical preparation is considered, a process of disinfection and shaping is thought of, and ‘bio' effectively means disinfection. While over the last 30 years there has been a huge uptake in the use of NiTi instrumentation techniques, it has not resulted in improved outcomes.^41^ Additional therapeutic means should be sought to improve outcomes of root canal treatment. Recently, it has been proposed to exploit dentine extracellular matrix components that exist naturally, which have been used in material-directed pulp-repair^42^^,^^43^ and apply such philosophies to treatment of periradicular disease.^44^ Although yet to be demonstrated, shifting thinking to more novel, biologically driven approaches are extremely important to explore.

## Conclusion

Endodontics is often mistakenly viewed as being a purely technical subject; however, a sound biological understanding is essential to execute effective treatment. Ongoing advancement in instrumentation techniques is making root canal preparation easier, allowing clinicians to achieve desired canal shape more quickly and enabling some canal systems previously deemed too difficult to negotiate and prepare, now more manageable. It is important not to overlook the irrigation phases during treatment, as the reduction in time spent on the mechanical stage of treatment may mean that disinfection of the canal could be inadequate. As the focus of development shifts from root canal preparation techniques to more biologically based treatment modalities for treating the root canal system, shaping of root canals may become less important.
